# Supplementary material for: Bed separation backfill to reduce surface cracking due to mining under thick and hard conglomerate: a case study
Source: R Soc Open Sci. 2019 Aug 21;6(8):190880. doi: 10.1098/rsos.190880 (PMC6731711; doi:10.1098/rsos.190880)
Supplement: Fig. 1 [file rsos190880supp2.doc]

| 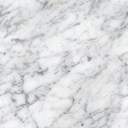 | **Quaternary system**  Surface soil layer with a thickness of 0~8 m. |
| --- | --- |
| 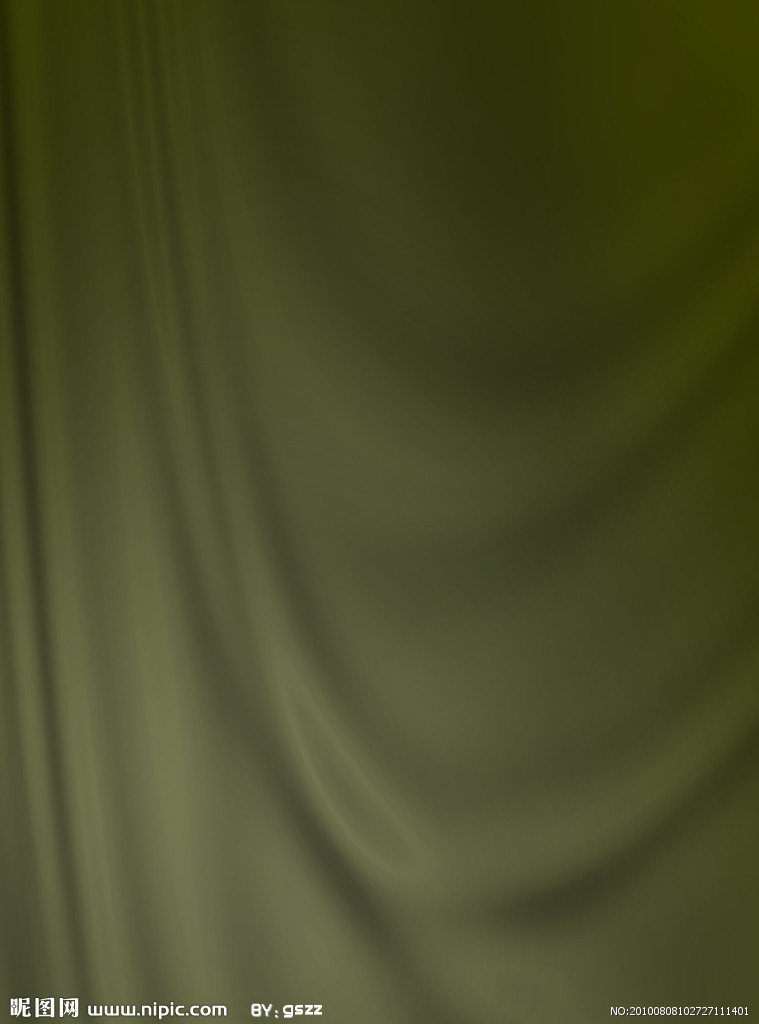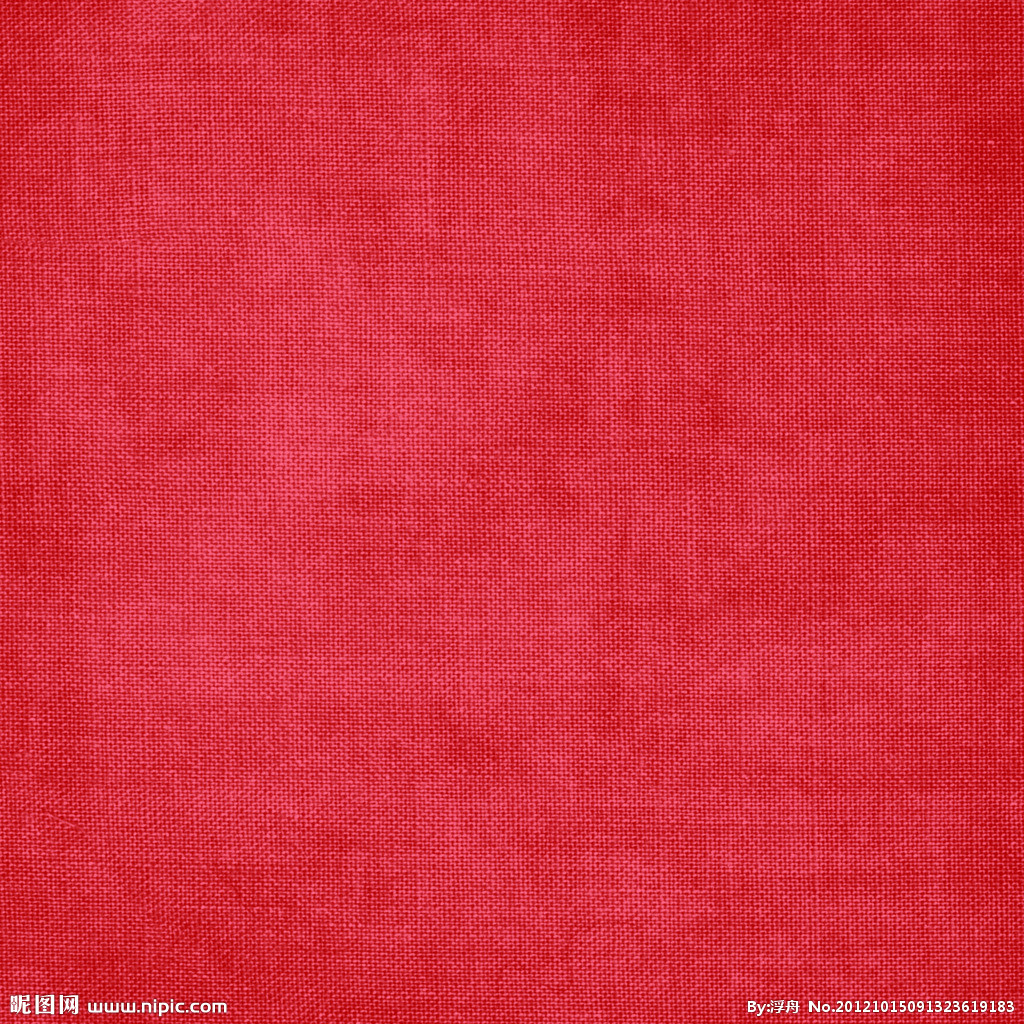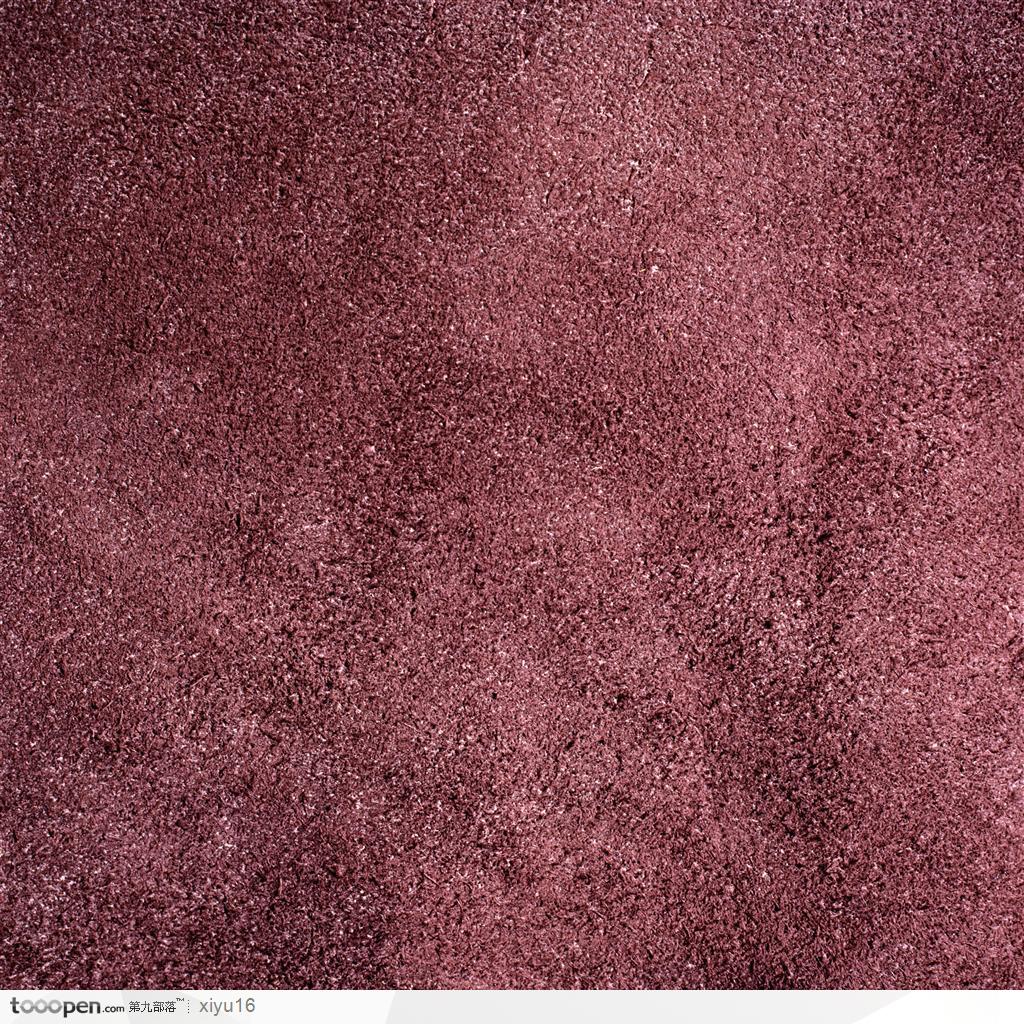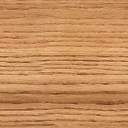 | **Tertiary system**  **Upper:** greyish-white, greyish-green and hard calcareous conglomerate with a large thickness of 400~800 m and a great uniaxial compressive strength of 62 MPa.  **Bottom:** red strata, reddish brown sandy clay or clayey siltstone mixed with thin conglomerate or pebbly sandstone, and with a thickness of 50 m. |
| 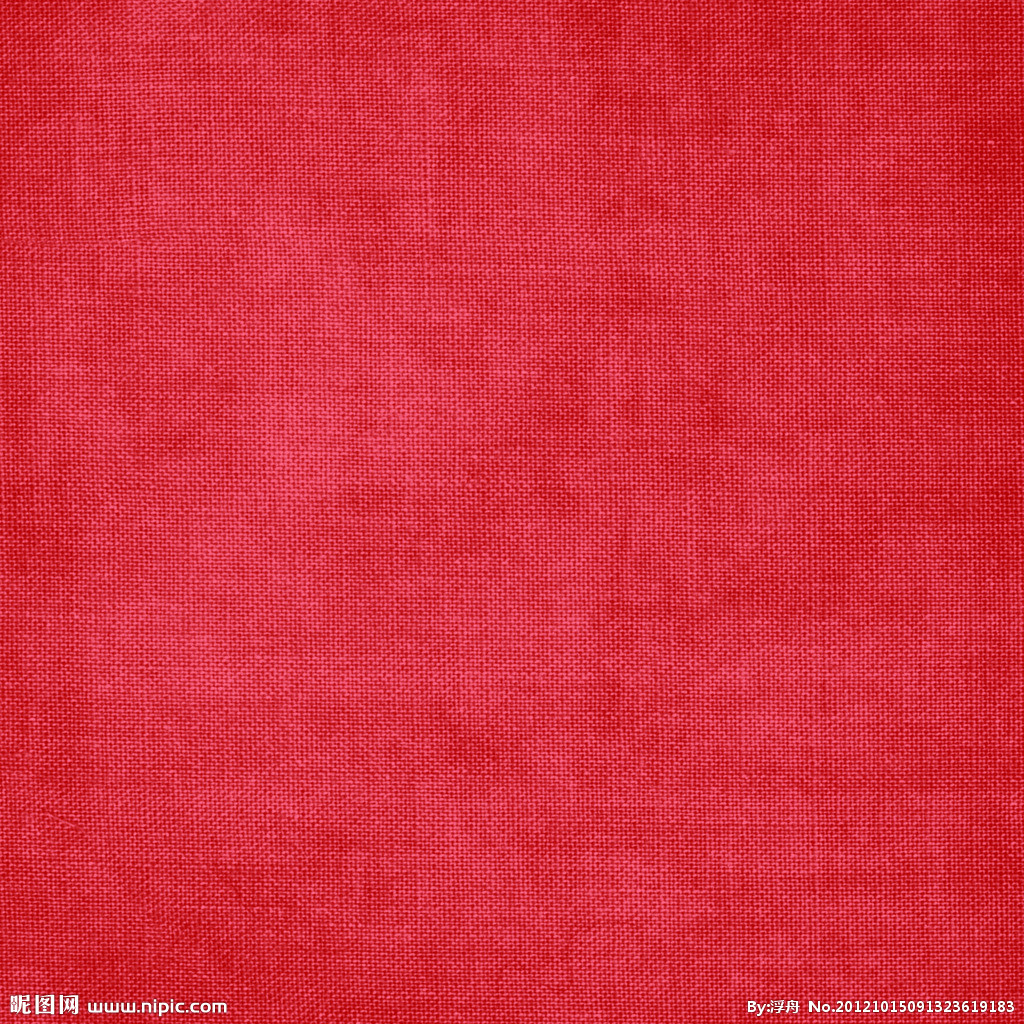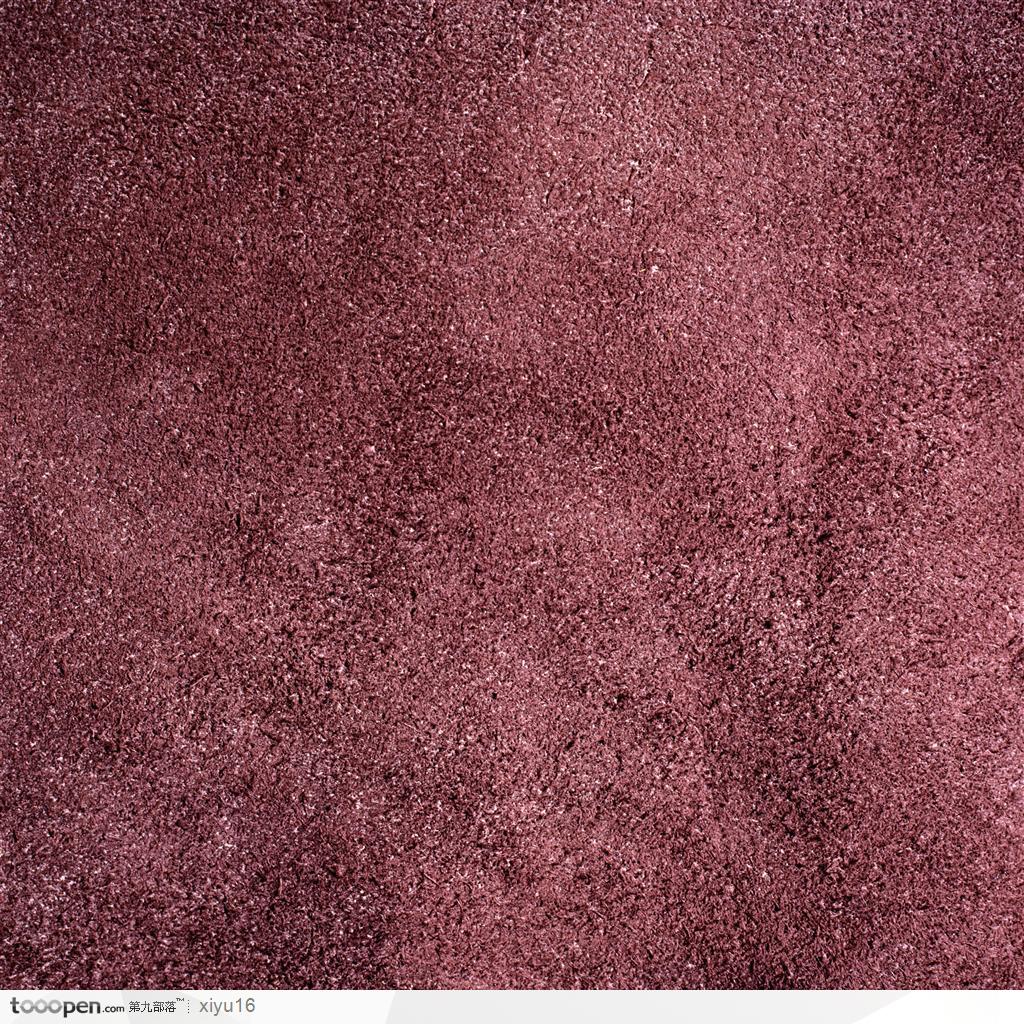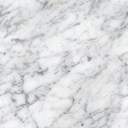 | **Shihezi Fm**  Varicolored clay stone and argillaceous conglomerate with a thickness of 0~154 m. |
| 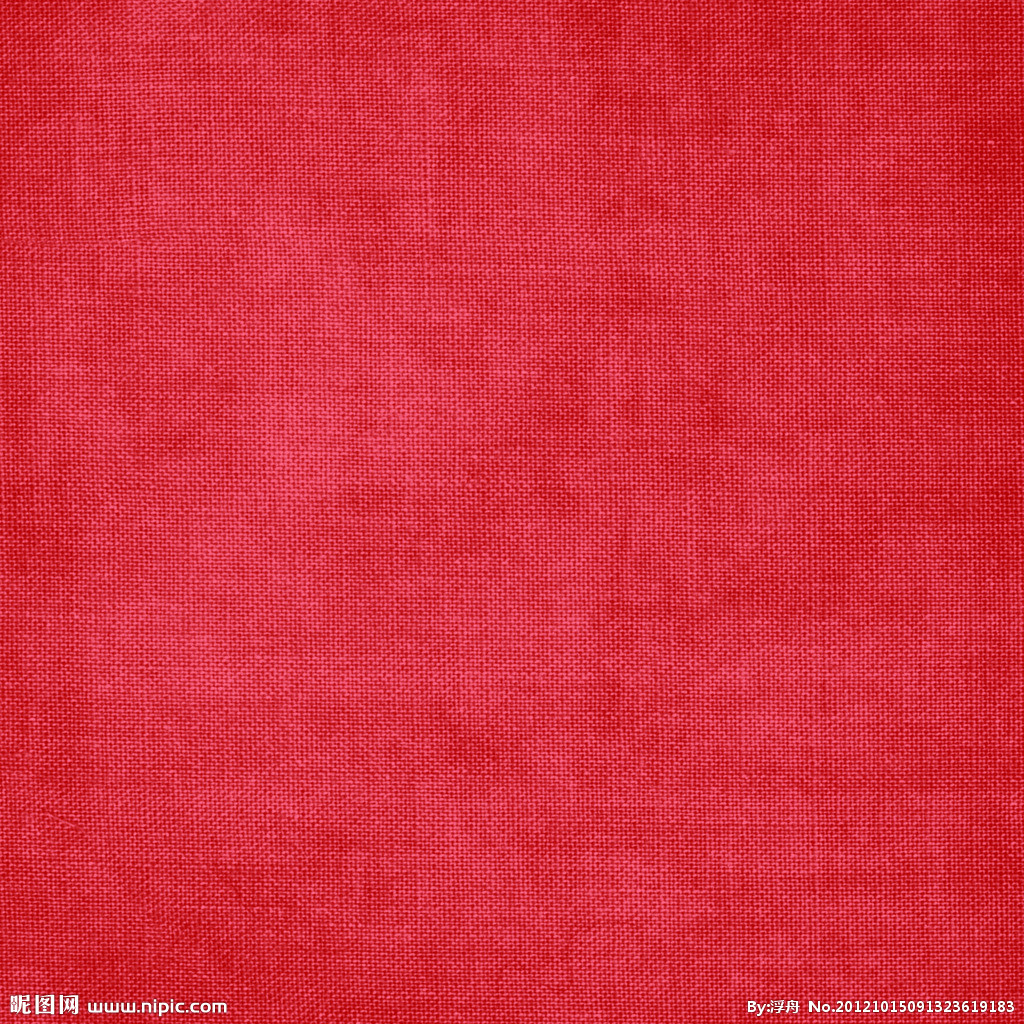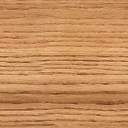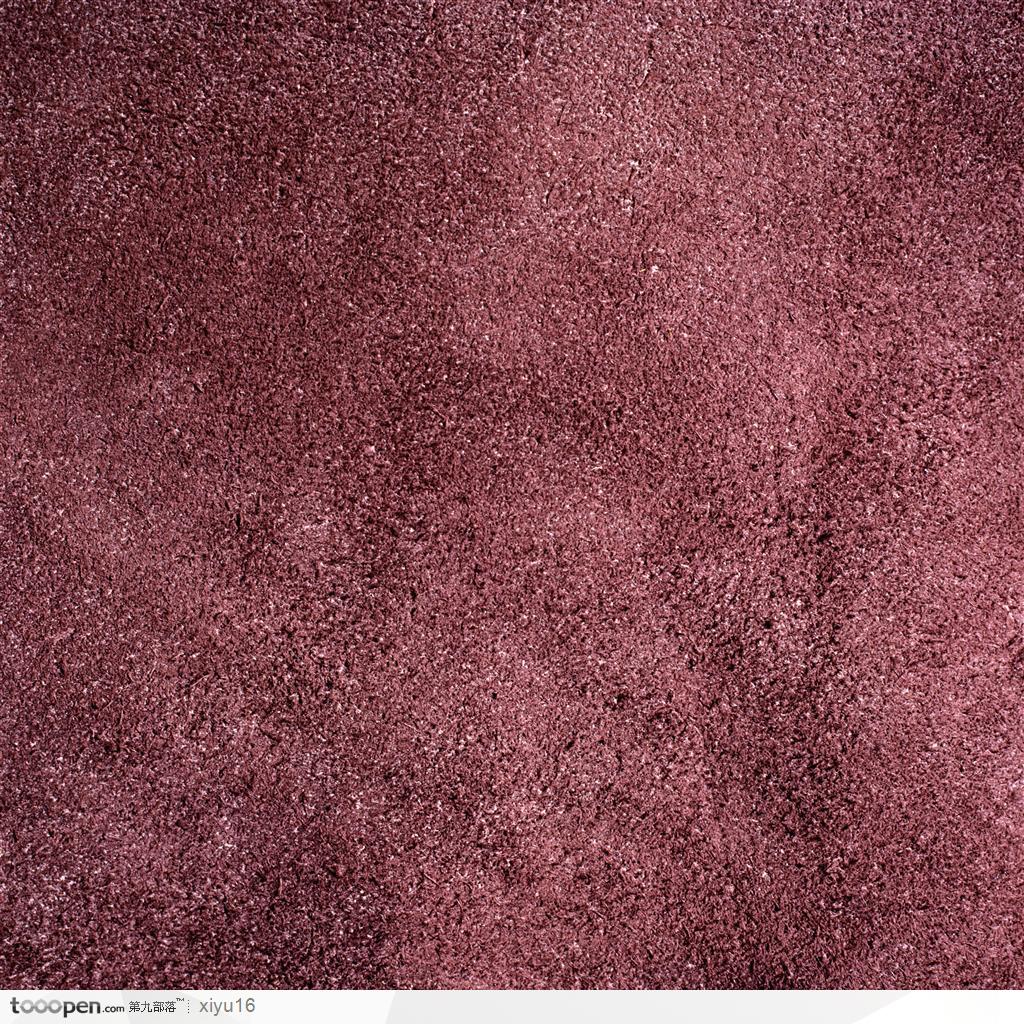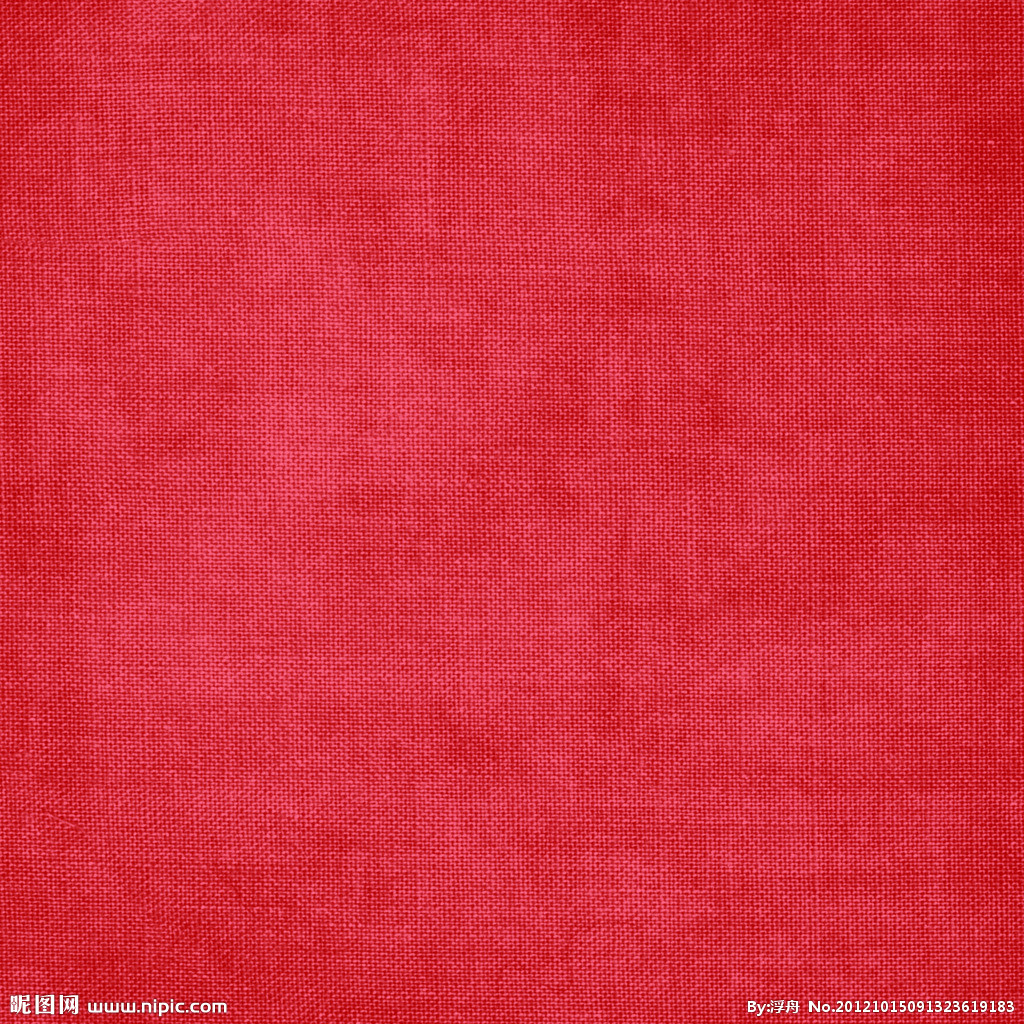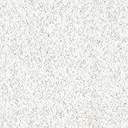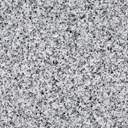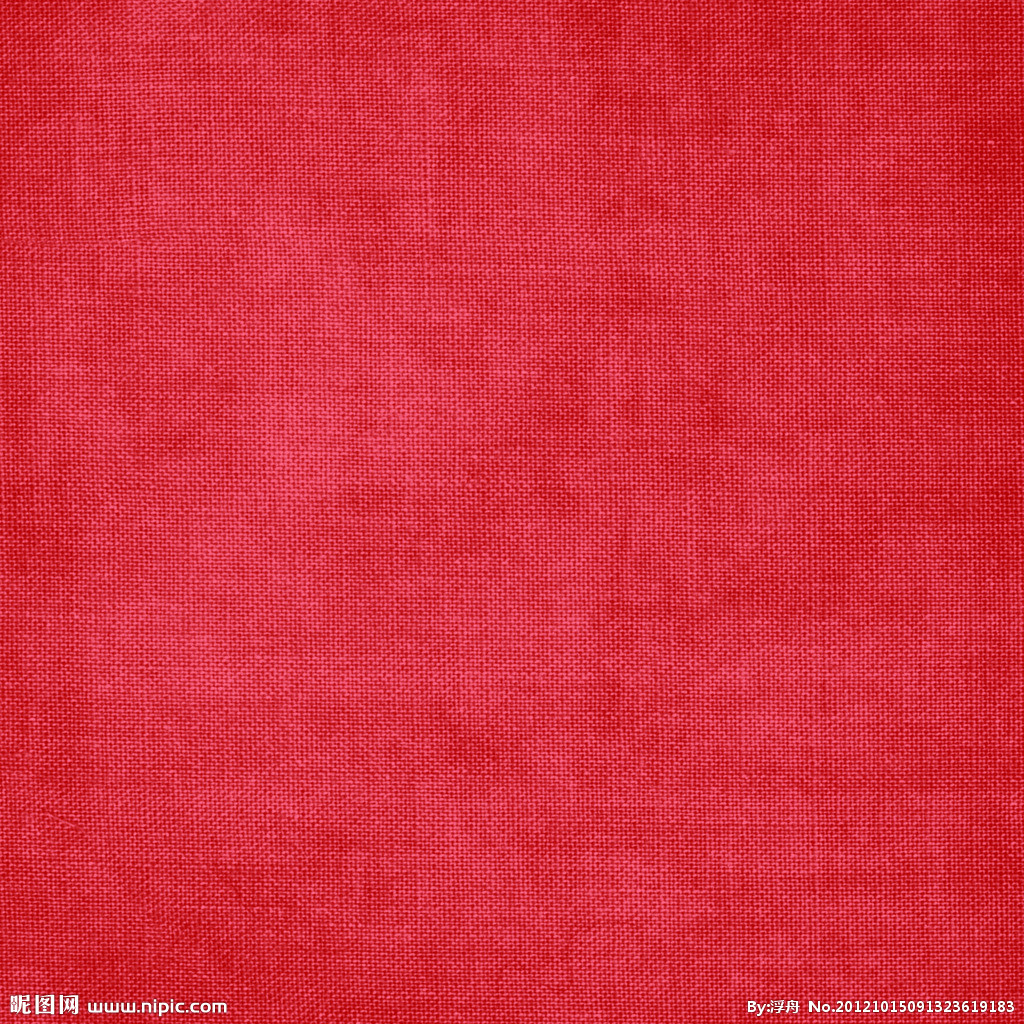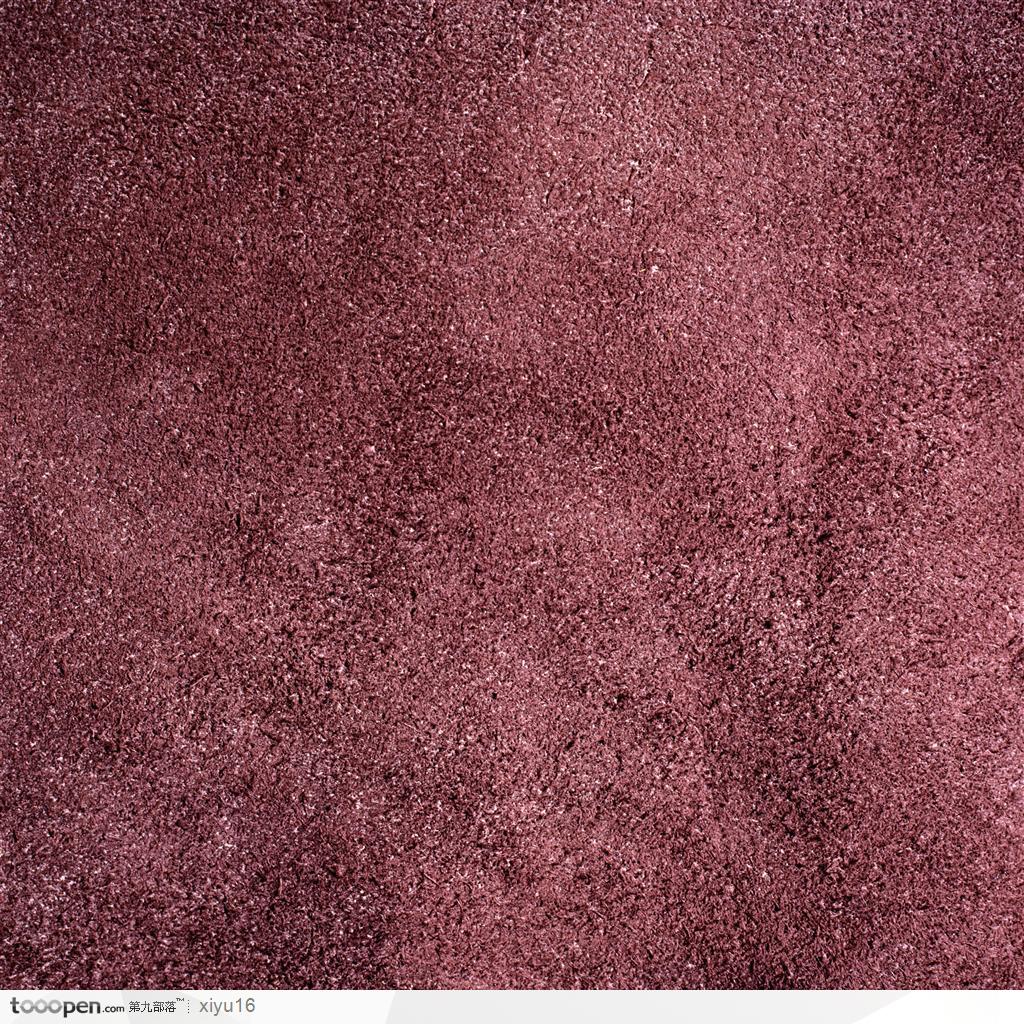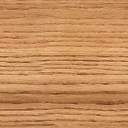 | **Stratigraphy of Shan-tung**  Sandstone, fine sandstone, a few of coarse sandstone and mudstone.  NO.4 and NO.6 coal seam are worth mining in the three coal seams. And NO.4 coal seam is main mining for coal. |

**Figure 1.** Comprehensive stratigraphic column of the Huafeng coal mine.
