# Supplementary material for: Bed separation backfill to reduce surface cracking due to mining under thick and hard conglomerate: a case study
Source: R Soc Open Sci. 2019 Aug 21;6(8):190880. doi: 10.1098/rsos.190880 (PMC6731711; doi:10.1098/rsos.190880)
Supplement: Fig. 11 [file rsos190880supp5.doc]

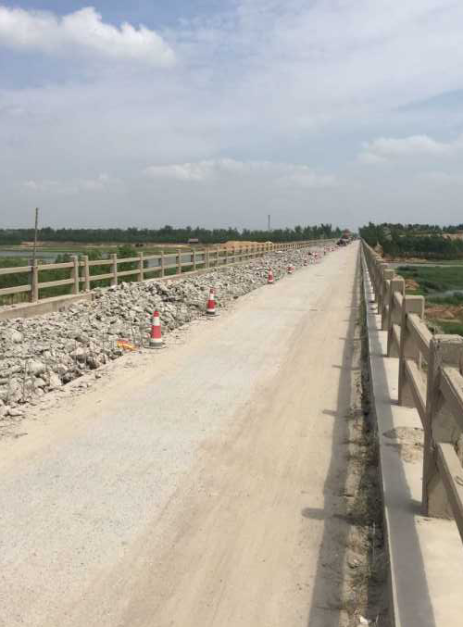

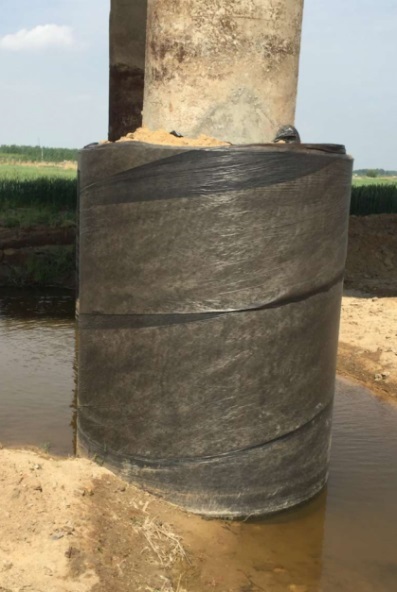


**Bridge deck pavement**

**New concrete**

(*a*)

(*b*)

**Figure 11.** Expand section for lower structure reinforcement. (a) Pave deck pavement, (b) Bridge pier.
