# Supplementary material for: Bed separation backfill to reduce surface cracking due to mining under thick and hard conglomerate: a case study
Source: R Soc Open Sci. 2019 Aug 21;6(8):190880. doi: 10.1098/rsos.190880 (PMC6731711; doi:10.1098/rsos.190880)
Supplement: Fig. 2 [file rsos190880supp6.doc]

(*a*)

(*b*)

**1.5 m**

**9.5 m**

**21.5 m**

**3.5 m**

**Figure 2.** Typical discontinuous surface deformations above the Huafeng coal mine. (a) Surface cracking [20], (b) Sunken pit induced by surface cracking.
