# Supplementary material for: Bed separation backfill to reduce surface cracking due to mining under thick and hard conglomerate: a case study
Source: R Soc Open Sci. 2019 Aug 21;6(8):190880. doi: 10.1098/rsos.190880 (PMC6731711; doi:10.1098/rsos.190880)
Supplement: Fig. 4 [file rsos190880supp8.doc]

**8 m**

**13 m**

**Figure 4.** Photograph of the Luli bridge
