# Supplementary material for: Bed separation backfill to reduce surface cracking due to mining under thick and hard conglomerate: a case study
Source: R Soc Open Sci. 2019 Aug 21;6(8):190880. doi: 10.1098/rsos.190880 (PMC6731711; doi:10.1098/rsos.190880)
Supplement: Fig. 5(b) [file rsos190880supp10.doc]

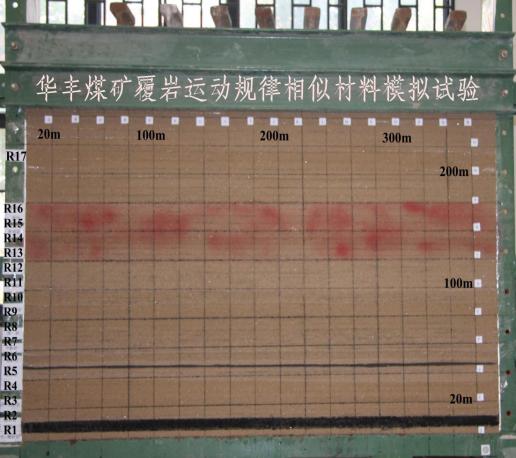


(*b*)

**Figure 5.** Experimental and physical models for the simulation test using similar material. (a) Schematic diagram of the experimental model, (b) Image of the physical model.
