# Supplementary material for: Bed separation backfill to reduce surface cracking due to mining under thick and hard conglomerate: a case study
Source: R Soc Open Sci. 2019 Aug 21;6(8):190880. doi: 10.1098/rsos.190880 (PMC6731711; doi:10.1098/rsos.190880)
Supplement: Fig. 6 [file rsos190880supp11.pdf]

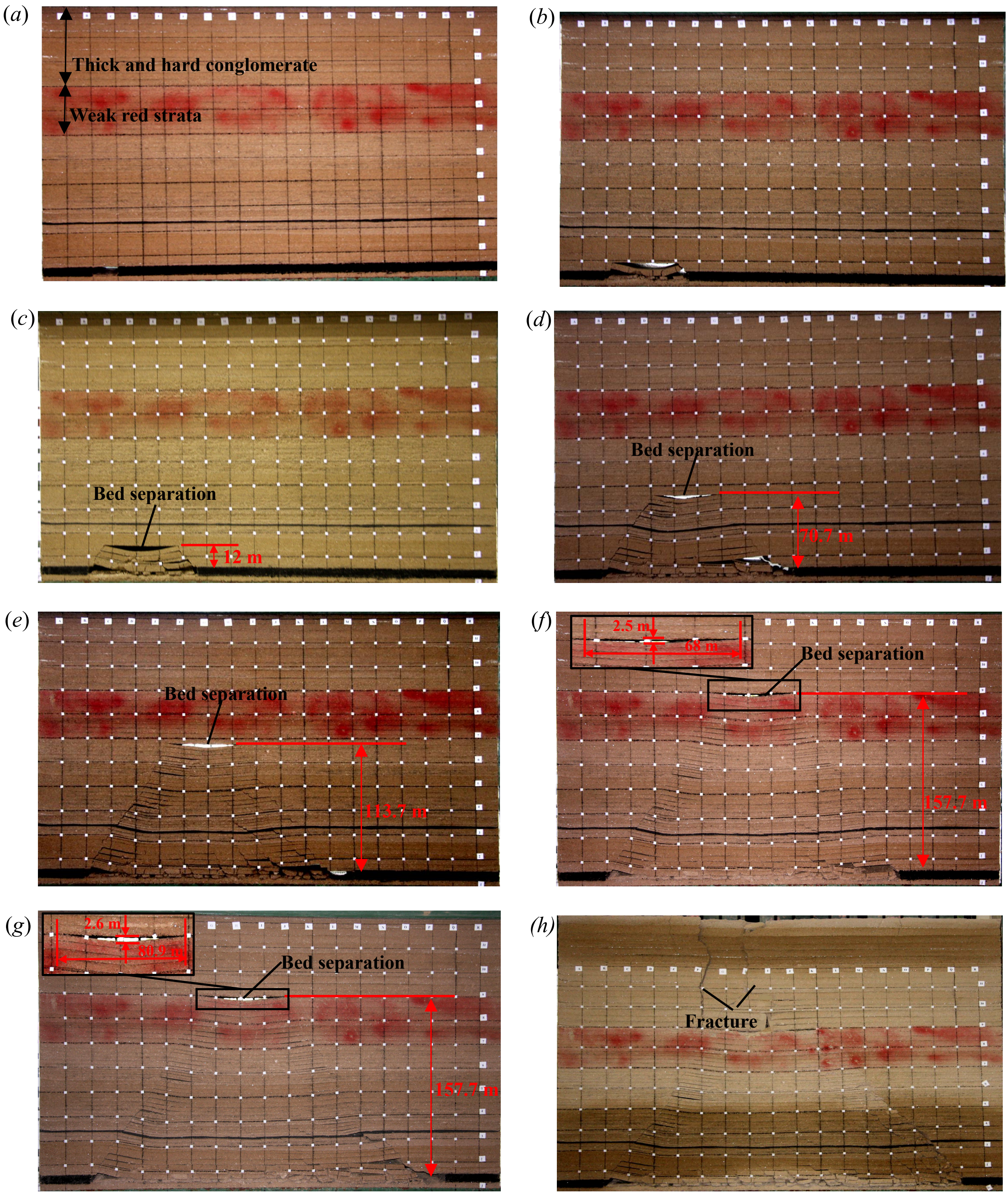

Figure 6. Photographs of the physical model showing movement of the overlying strata. (a) open-off cut, (b) advance distance of 40 m, (c) advance distance of 70 m, (d) advance distance of 150 m, (e) advance distance of 220 m, (f) advance distance of 250 m, (g) advance distance of 290 m, (h) advance distance of 320 m.
