# Supplementary material for: Bed separation backfill to reduce surface cracking due to mining under thick and hard conglomerate: a case study
Source: R Soc Open Sci. 2019 Aug 21;6(8):190880. doi: 10.1098/rsos.190880 (PMC6731711; doi:10.1098/rsos.190880)
Supplement: Fig. 7 [file rsos190880supp12.doc]

(*a*)

(*b*)

**Conglomerate**

**Red strata**

**Red strata**

**Conglomerate**

**Figure 7.** Ultrasonic images of borehole pre- and post-mining [20]. (a) Before coal mining, (b) After coal mining.
