# Supplementary material for: Bed separation backfill to reduce surface cracking due to mining under thick and hard conglomerate: a case study
Source: R Soc Open Sci. 2019 Aug 21;6(8):190880. doi: 10.1098/rsos.190880 (PMC6731711; doi:10.1098/rsos.190880)
Supplement: Table 1 [file rsos190880supp15.doc]

**Table 1. Proportion, materials, and layout order of the model strata [21].**

| Stratum | Lithology | Actual thickness (m) | Thickness in simulated model (cm) | Layered number | Layered thickness (cm) | Bulk density of similar materials (g/cm^3^) | Material consumption (kg) | | | | Uniaxial compressive strength of the prototype (MPa) | Uniaxial compressive strength  of the model (KPa) |
| --- | --- | --- | --- | --- | --- | --- | --- | --- | --- | --- | --- | --- |
|  |  |  |  |  |  |  | Sand | Lime | Gypsum | Water |  |  |
| R17 | Thick conglomerate | 144 | 72 | 18 | 4 | 1.45 | 28.44 | 1.78 | 1.78 | 2.90 | 62.0 | 105.31 |
| R16 | Clayey siltstone | 8.35 | 4.2 | 2 | 2.1 | 1.5 | 15.45 | 1.16 | 0.77 | 1.58 | 32.2 | 54.69 |
| R15 | Mudstone | 15.6 | 7.8 | 3 | 2.6 | 1.5 | 19.13 | 1.67 | 0.72 | 1.96 | 25.6 | 43.48 |
| R14 | Clayey siltstone | 20.0 | 10 | 5 | 2 | 1.5 | 37.01 | 2.78 | 1.84 | 3.78 | 32.2 | 54.69 |
| R13 | Mudstone | 16.55 | 8.3 | 5 | 1.66 | 1.5 | 20.29 | 1.78 | 0.77 | 2.09 | 25.6 | 43.48 |
| R12 | Medium grain sandstone | 3.85 | 1.9 | 2 | 0.95 | 1.6 | 7.45 | 0.47 | 0.47 | 0.76 | 36.6 | 62.17 |
| R11 | Siltstone | 4.6 | 2.3 | 2 | 1.15 | 1.5 | 8.46 | 0.63 | 0.42 | 0.87 | 43.4 | 73.72 |
| R10 | Medium grain sandstone | 28.85 | 14.4 | 8 | 1.8 | 1.6 | 55.83 | 3.52 | 3.52 | 5.70 | 36.6 | 62.17 |
| R9 | Mudstone | 6.6 | 3.3 | 2 | 1.65 | 1.5 | 8.09 | 0.71 | 0.30 | 0.83 | 25.6 | 43.48 |
| R8 | Medium grain sandstone | 3.95 | 2 | 2 | 1.0 | 1.6 | 7.85 | 0.49 | 0.49 | 0.80 | 36.6 | 62.17 |
| R7 | Siltstone | 1.17 | 1.5 | 2 | 0.75 | 1.5 | 5.61 | 0.42 | 0.28 | 0.58 | 43.4 | 73.72 |
| R6 | Coal seam 6 | 1.20 | 0.6 | 1 | 0.6 | 1.35 | 3.98 | 0.34 | 0.15 | 0.41 | 8.42 | 14.30 |
| R5 | Siltstone | 5.29 | 2.6 | 2 | 1.3 | 1.5 | 9.73 | 0.72 | 0.48 | 1.00 | 43.4 | 73.72 |
| R4 | Sandy mudstone | 5.25 | 2.6 | 2 | 1.3 | 1.5 | 9.56 | 0.84 | 0.36 | 0.98 | 23.2 | 39.41 |
| R3 | Mid-fine grained sandstone | 20 | 10 | 5 | 2 | 1.6 | 15.69 | 0.98 | 0.98 | 1.61 | 48.48 | 82.34 |
| R2 | Siltstone | 2.6 | 1.3 | 1 | 1.3 | 1.5 | 4.78 | 0.36 | 0.24 | 0.49 | 43.4 | 73.72 |
| R1 | Coal seam 4 | 6.41 | 3.2 | 1 | 3.2 | 1.35 | 21.19 | 1.85 | 0.79 | 2.17 | 10.4 | 17.66 |
| Floor | Fine sandstone | 10 | 5 | 2 | 2.5 | 1.5 | 19.55 | 1.23 | 1.23 | 2.01 | 51.2 | 86.96 |
