# Supplementary material for: Bed separation backfill to reduce surface cracking due to mining under thick and hard conglomerate: a case study
Source: R Soc Open Sci. 2019 Aug 21;6(8):190880. doi: 10.1098/rsos.190880 (PMC6731711; doi:10.1098/rsos.190880)
Supplement: Table 2 [file rsos190880supp16.doc]

**Table 2.** Plane map distances from drilling holes to bridges and working faces.

| Drill hole | To the Luli Bridge | To the trend line of Luli Bridge | To the lower roadway of 1612 working face | To the lower roadway of 1411 working face |
| --- | --- | --- | --- | --- |
| 10-1 | 510 m | 260 m | 34 m | 130 m |
| 10-2 | 470 m | 10 m | 32 m | 127 m |
| 10-3 | 630 m | 140 m | 80 m | 40 m |
